# Efficacy and Safety of Xinyue Capsule for Coronary Artery Disease after Percutaneous Coronary Intervention: A Systematic Review and Meta-Analysis of Randomized Clinical Trials

**DOI:** 10.1155/2021/6695868

**Published:** 2021-04-08

**Authors:** Zhonghui Jiang, Hua Qu, Ying Zhang, Fan Zhang, Wenli Xiao, Dazhuo Shi, Zhuye Gao, Keji Chen

**Affiliations:** ^1^Department of Cardiology, Xiyuan Hospital, China Academy of Chinese Medical Sciences, Beijing 100091, China; ^2^National Clinical Research Center for Chinese Medicine Cardiology, Beijing 100091, China

## Abstract

To evaluate the efficacy and safety of Xinyue capsule (XYC) in the treatment of coronary artery disease (CAD) after percutaneous coronary intervention (PCI), databases including MEDLINE, EMBASE (Ovid), PubMed, Google Scholar, Cochrane Central Register of Controlled Trials (CENTRAL), China National Knowledge Infrastructure database (CNKI), Wanfang, and VIP were searched to identify randomized controlled trials (RCTs) on XYC in CAD after PCI published before October 2020. Data extraction, methodological quality assessment, and data analysis were performed according to the Cochrane standard. Dichotomous data were shown as risk ratios (RRs) with a 95% confidence interval (CI). All analyses were done with Review Manager, version 5.3. The quality of evidence was assessed by the Grading of Recommendations Assessment, Development and Evaluation (GRADE) approach. A total of 9 related studies from 166 related articles were identified, which included 2979 patients. Compared with conventional treatment alone (or placebo plus), XYC decreased cardiovascular events [RR = 0.37, 95% CI (0.27, 0.51), *I*^2^ = 0%] (nonfatal myocardial infarction [RR = 0.26, 95% CI (0.10, 0.70), *I*^2^ = 0%], revascularization [RR = 0.38, 95% CI (0.24, 0.61), *I*^2^ = 0%], and rehospitalization due to ACS [RR = 0.48, 95% CI (0.33, 0.68), *I*^2^ = 0%]) and improved cardiac function (LVEF [RR = 6.93, 95% CI (4.99, 8.87), *I*^2^ = 81%], LVEDV [RR = −4.07, 95% CI (−5.61, −2.54), *I*^2^ = 7%], and LVESV [RR = −4.32, 95% CI (−5.90, −2.74), *I*^2^ = 50%]) in patients after PCI. In addition, XYC reduced serum NT-pro-BNP [RR = −126.91, 95% CI (−231.51, −22.31), *I*^2^ = 69%]. However, XYC had little effect on cardiovascular death [RR = 0.47, 95% CI (0.13, 1.68), *I*^2^ = 0%], stroke [RR = 0.52, 95% CI (0.23, 1.20), *I*^2^ = 0%], heart failure [RR = 0.53, 95% CI (0.24, 1.20), *I*^2^ = 0%], and quality of life [RR = −1.37, 95% CI (−4.97, 2.22), *I*^2^ = 93%]. Thus, this meta-analysis suggests that XYC has potential advantages in reducing the occurrence of cardiovascular events after PCI, improving cardiac function, and reducing serum NT-pro-BNP. This potential benefit requires a high-quality RCT to assess.

## 1. Introduction

Coronary heart disease (CHD) is a common and frequently occurring disease of the cardiovascular system that seriously endangers human health. It is the leading cause of death worldwide and contributes considerably to its morbidity [1, 2]. Data from the Global Burden of Disease Study 2015 show that in 2015 there were 422.27 million cases of cardiovascular disease (CVD) (95% CI: 415.53–427.87 million) and 17.92 million CVD-related deaths (95% CI: 17.59–18.28 million CVD) [3]. The number of deaths from heart disease is projected to reach 23.3 million by 2030 [4]. Approximately 50% of the mortality associated with cardiovascular disease is due to CHD [5]. A study was conducted in collaboration with the United States, and based on the current prevalence of cardiovascular risk factors in China, it was estimated that there will be an additional 21.3 million cardiovascular patients and 7.7 million cardiovascular deaths in China between 2010 and 2030 [6]. The high morbidity and mortality of CHD will bring a great burden to the global society.

With the development of coronary interventional therapy and surgical techniques, the mortality rate of CHD has decreased significantly, but there is still a high incidence of major adverse cardiac events (MACEs) after stenting (2.1–19%) [7–9]. Postoperative restenosis, stent thrombosis, heart failure, and arrhythmia, for example, are still great challenges for western medicine [10]. The combination of traditional Chinese and western medicine has potential advantages in the treatment of cardiovascular events after interventional therapy for CHD.

Xinyue capsule (XYC) is a patented Chinese herbal medicine that has been used to treat CHD for over a decade in China. XYC was approved by the Chinese Food and Drug Administration (Z20030073) for the treatment of coronary artery disease (CAD) in 2005. Panax quinquefolius saponins (PQSs) are major bioactive components in XYC, each capsule contains 100 mg PQS, and XYC is taken orally, 2 capsules per time, 3 times a day [11]. Plenty of previous studies have shown that XYC could relieve myocardial ischemia, alleviate clinical symptoms, improve myocardial reperfusion after percutaneous coronary intervention (PCI), reduce recurrent angina, and exhibit other pharmacological functions [12–14], but there was little solid evidence for its efficacy and safety. To provide a comprehensive synthesis of effect estimates and quality of evidence for clinical application, a systematic review and meta-analysis of randomized trials of XYC for CAD after PCI was conducted.

## 2. Materials and Methods

### 2.1. Search Strategies

The meta-analysis of the clinical trial was constructed following the PRISMA guidelines [15, 16]. Results were obtained from completed, published, randomized trials of XYC for CAD after PCI. Databases searched include MEDLINE, EMBASE (Ovid), PubMed, Google Scholar, Cochrane Central Register of Controlled Trials (CENTRAL), China National Knowledge Infrastructure database (CNKI), Wanfang, and VIP from database inception to October 2020. Two reviewers (Zhonghui Jiang and Hua Qu) independently searched through the above electronic databases. MeSH terms and free words were used reasonably through the characteristics of literature databases. The detailed searching strategies are shown in Appendices 1 and 2.

### 2.2. Eligibility Criteria

#### 2.2.1. Types of Studies

Randomized controlled trials (RCTs) of XYC in the treatment of coronary heart disease after PCI without limitation of published language or blind methods were included. The original text (including efficacy evaluation indicators) or accurate data required for analysis can be obtained. The conference papers were excluded.

#### 2.2.2. Types of Participants

Patients had been diagnosed with CAD by coronary angiography and had successfully undergone PCI. Age, sex, and race were not subject to appropriate restrictions.

#### 2.2.3. Types of Interventions

The treatment group was given XYC alone or combined with other drugs on the basis of conventional treatment (except proprietary Chinese medicines with similar functions), and the control group was given placebo or conventional treatment. Conventional western medicines include angiotensin-converting enzyme inhibitors, antiplatelet aggregation drugs, angiotensin receptor blockers, beta-blockers, calcium antagonists, statins, and nitrates.

#### 2.2.4. Types of Outcome Measures

*(1)* Primary outcomes are as follows: ① primary cardiovascular events: cardiac death, nonfatal myocardial infarction, revascularization, heart failure, stroke, and rehospitalization due to acute coronary syndrome (ACS); ② heart function: left ventricular ejection fraction (LVEF), left ventricular end-diastolic volume (LVEDV), left ventricular end-systolic volume (LVESV), left ventricular end-diastolic dimension (LVEDD), interventricular septum thickness (IVST), and left ventricular posterior wall thickness (LVPWT).

*(2)* Secondary outcomes are as follows: ① serum N-terminal pro-B-type natriuretic peptide (NT-pro-BNP); ② quality of life: quality of life was assessed using a 36-item Short-Form Health Survey (SF-36); ③ adverse reaction.

### 2.3. Data Extraction and Analysis

Two investigators (Zhonghui Jiang and Ying Zhang) independently screened data including authors, title, publication year, inclusion and exclusion criteria, sample size, study design, baseline patients' features (age, gender, and diagnosis criteria), intervention measures (dosage, usage, and treatment duration) for the treatment group and control group, endpoint definitions, and effect measured. Any disagreements were resolved through discussion with a third investigator (Keji Chen). The authors were contacted where possible if the information was incomplete or unclear. Data was managed in accordance with the principles of the intended treatment.

### 2.4. Risk of Bias in Individual Studies

The Cochrane Handbook for Systematic Reviews of Interventions (updated September 2009) was used to assess the risk of bias. Three reviewers (Zhonghui Jiang, Fan Zhang, and Wenli Xiao) independently assessed seven areas of bias for each outcome: (1) randomization process, (2) allocation hiding, (3) incomplete or missing outcome data, (4) deviation from expected interventions, (5) application of the blind method in research, (6) measurement of results, and (7) selection of reporting outcome. Disagreements were resolved through discussion.

### 2.5. Data Synthesis and Analysis

In this meta-analysis, the clinical heterogeneity among study results was analyzed by Chi-squared (*χ*^2^) test, the test level was *α* = 0.1, and *I*^2^-test was used to measure the heterogeneity. Where results are homogeneous (*P* > 0.1, *I*^2^ < 50%), the fixed-effects model was used for meta-analysis. If heterogeneity existed among the study results (*P* ≤ 0.1, *I*^2^ ≥ 50%), the random-effects model was used for meta-analysis. The *Z* test was used for the combined effect value, and the test level was *α* = 0.05. To estimate the size of the comprehensive effect, sensitivity analysis was performed on the meta-analysis again after eliminating individual studies. Publication bias was visually judged by drawing funnel plots and quantitative detection by using the Egger regression method, and the test level was *α* = 0.05. Statistical analyses were performed using the Review Manager, version 5.3. There is currently no registered protocol for this meta-analysis.

## 3. Results

### 3.1. Study Selection and Characteristics

**Figure 1:**
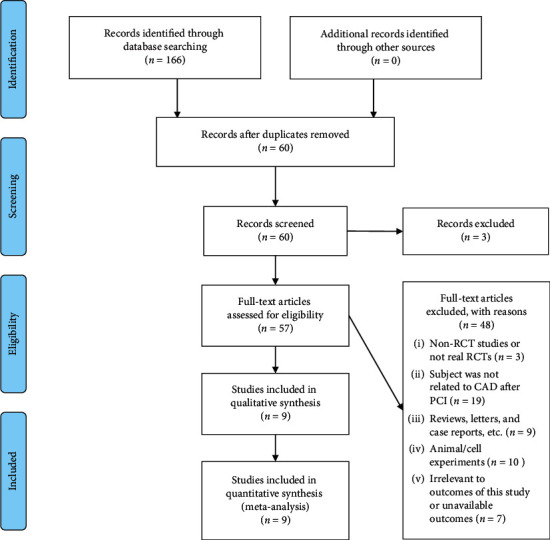
The process of the literature search and study selection.

As shown in Figure 1, we identified 166 potentially relevant articles from the database. After removing 106 duplicate studies, 60 studies were selected for further examination. After filtering titles and abstracts, 3 studies were excluded because of apparent disqualification. Of the remaining 57 studies, after the full-text screening, a further 48 studies were excluded. Finally, 9 studies were included in the meta-analysis.

### 3.2. Characteristics of Included Studies

The characteristics of the 9 included trials are shown in Table 1. Between 2010 and 2020, 9 suitable randomized controlled trials [17–25] involving 2979 participants were published, with an average sample size of 331 for each trial (ranging from 64 to 1054).

### 3.3. Methodological Quality Assessment

#### 3.3.1. Random Sequence Generation Methods

All included studies were RCTs and mentioned the word “randomization,” but 1 study [25] only mentioned the word “random” and did not report it in detail. Six studies [19–24] were of low risk and identified specific random allocation methods, including computer-generated random numbers and field stratification and random number table. However, 2 studies [17, 18] were high-risk and adopted randomization of treatment sequence.

#### 3.3.2. Allocation Concealment

One study [22] used a randomized drug distribution scheme with drug number concealment, and 3 studies [20, 21, 23] adopted an opaque envelope for random allocation scheme concealment, which were considered as low risk, while the remaining 5 studies were not mentioned.

#### 3.3.3. Blinding

Two studies [22, 25] used placebo as control and blinded both participants and personnel, which were assessed as having a low risk. One study [23] was open-label and was assessed as high risk. The outcomes of 4 studies [20, 22, 23, 25] were evaluated by a third party, so were all blind and low-risk studies. The remaining 5 studies were unclear.

#### 3.3.4. Data Integrity

All study data were complete and were evaluated as having a low risk.

#### 3.3.5. Other

**Figure 2:**
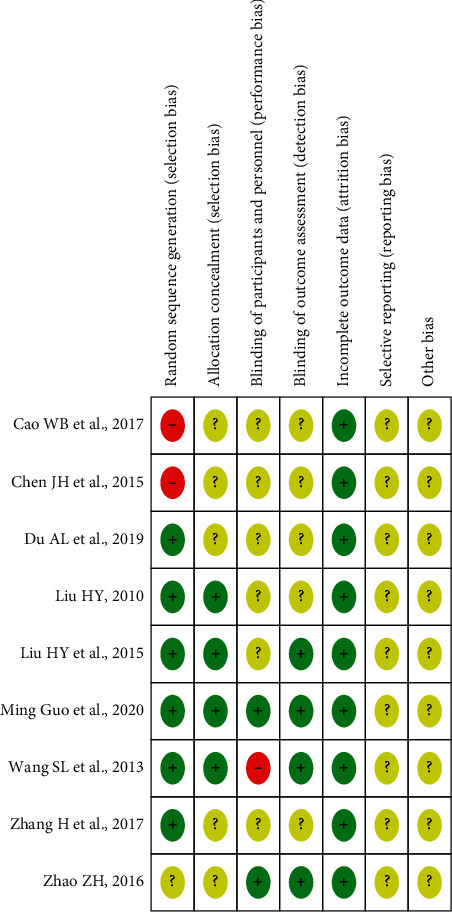
Summary of bias risk assessment for included studies.

**Figure 3:**
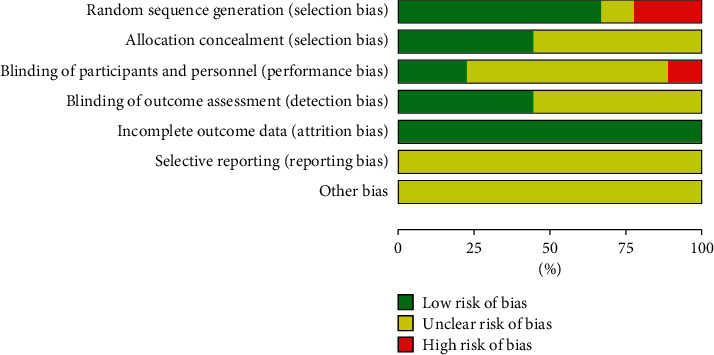
Graph of bias risk assessment for included studies.

The results of selective reporting and other biases are not clear. Risk assessments of included study bias are shown in Figures 2 and 3.

### 3.4. Primary Outcomes

#### 3.4.1. Primary Cardiovascular Events

**Figure 4:**
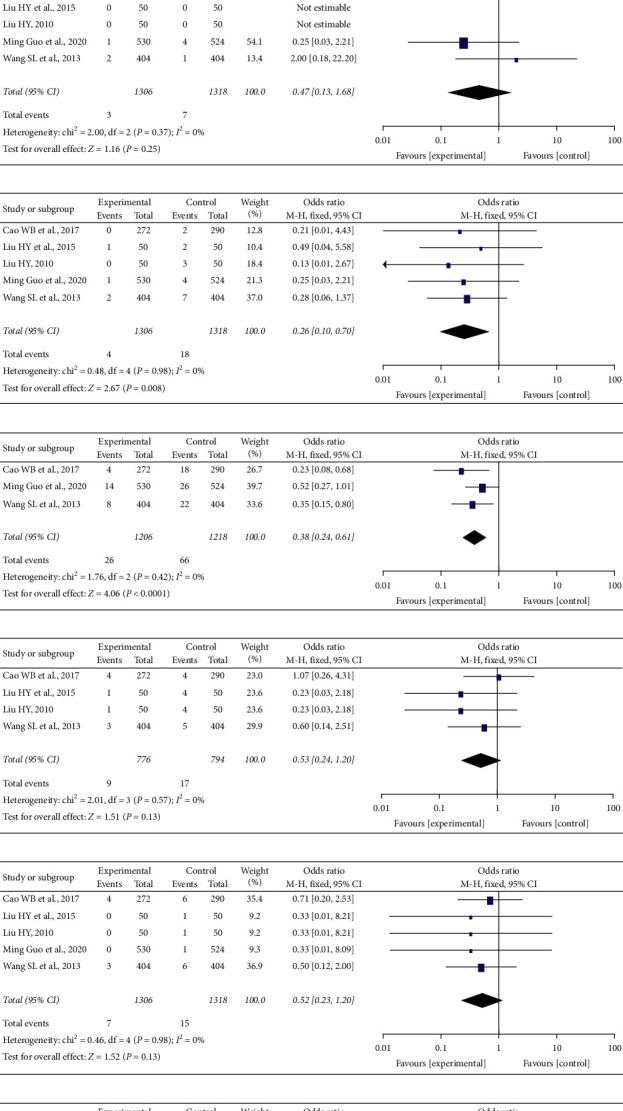
Primary cardiovascular events.

Based on conventional western medicine treatment, XYC alone or combined with other western medicines could effectively reduce the risk of cardiovascular events. As shown in Figure 4(a), a total of 5 trials [17, 20–23] including 2624 patients (XYC group: 1306, control group: 1318) reported the occurrence of total clinical events, indicating that the XYC group had better efficacy on lowering the cardiovascular events compared with the control group [RR = 0.37, 95% CI (0.27, 0.51), *I*^2^ = 0%].

*(1) Cardiac Death*. Five studies [17, 20–23] reported cardiac death. There was no evidence that the XYC group was better than the control group [RR = 0.47, 95% CI (0.13, 1.68), *I*^2^ = 0%], as shown in Figure 4(b).

*(2) Nonfatal Myocardial Infarction*. Five studies [17, 20–23] reported nonfatal myocardial infarction; the results showed that the XYC group had a lower rate of reinfarction [RR = 0.26, 95% CI (0.10, 0.70), *I*^2^ = 0%] (Figure 4(c)).

*(3) Revascularization*. Three studies [17, 22, 23] described revascularization; the meta-analysis showed that the XYC group was better at lowering revascularization than the control group [RR = 0.38, 95% CI (0.24, 0.61), *I*^2^ = 0%] (Figure 4(d)).

*(4) Heart Failure*. Four studies [17, 20, 21, 23] reported heart failure; the results showed that the difference between the two groups was not statistically significant [RR = 0.53, 95% CI (0.24, 1.20), *I*^2^ = 0%] (Figure 4(e)).

*(5) Stroke*. The incidence of stroke was observed in 5 of the studies [17, 20–23]. There was no statistical significance in stroke occurrence between the two groups [RR = 0.52, 95% CI (0.23, 1.20), *I*^2^ = 0%] (Figure 4(f)).

*(6) Rehospitalization due to ACS*. Three studies [17, 22, 23] reported rehospitalization due to ACS. The meta-analysis showed the XYC group significantly reduced the incidence of rehospitalization due to ACS, compared with the control group [RR = 0.48, 95% CI (0.33, 0.68), *I*^2^ = 0%] (Figure 4(g)).

#### 3.4.2. Heart Functions

**Figure 5:**
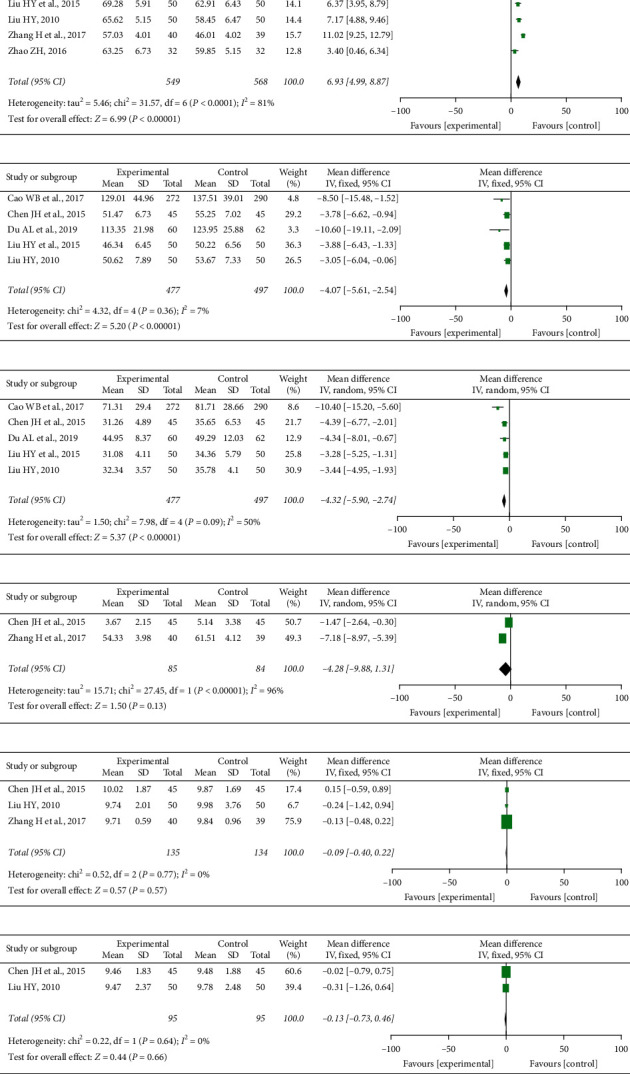
Heart function.

*(1) LVEF*. Seven studies [17–21, 24, 25] reported the LVEF, including 549 patients in the XYC group and 568 patients in the control group. The meta-analysis showed that the XYC group could increase the LVEF significantly [RR = 6.93, 95% CI (4.99, 8.87), *I*^2^ = 81%] (Figure 5(a)). Due to high heterogeneity, the sensitivity of LVEF was analyzed. When the two studies [17, 24] were deleted, there was no significant change in the results [RR = 5.74, 95% CI (4.60, 6.88), *P* < 0.00001].

*(2) LVEDV*. Five studies [17–21] reported the LVEDV, involving 974 patients. The studies showed that the XYC group could effectively regulate LVEDV compared with the control group [RR = −4.07, 95% CI (−5.61, −2.54), *I*^2^ = 7%] (Figure 5(b)).

*(3). LVESV*. Five studies [17–21] reported the LVESV, involving 974 patients. The studies showed that the XYC group could effectively regulate LVESV compared with the control group [RR = −4.32, 95% CI (−5.90, −2.74), *I*^2^ = 50%] (Figure 5(c)). Due to high heterogeneity, the sensitivity of LVESV was analyzed. When one of the studies [17] was deleted, there was no significant change in the results [RR = −3.64, 95% CI (−4.67, −2.62), *P* < 0.00001].

*(4) LVEDD*. Two studies [18, 24] reported the LVEDD, involving 169 patients. There was no statistical significance in LVEDD [RR = −4.28, 95% CI (−9.88, 1.31), *I*^2^ = 96%] (Figure 5(d)). Due to the small number of literature available, sensitivity analysis could not be carried out.

*(5) IVST*. Three studies [18, 21, 24] reported the IVST, totaling 269 patients. There was no statistical significance in IVST between groups [RR = −0.09, 95% CI (−0.40, 0.22), *I*^2^ = 0%] (Figure 5(e)).

*(6) LVPWT*. Two studies [18, 21] reported the LVPWT, including 190 patients. There was no statistical significance in LVPWT between groups [RR = −0.13, 95% CI (−0.73, 0.46), *I*^2^ = 0%] (Figure 5(f)).

### 3.5. Secondary Outcomes

#### 3.5.1. Serum NT-pro-BNP

**Figure 6:**
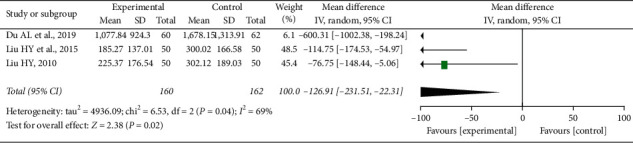
NT-pro-BNP.

Compared with the control group, meta-analysis results of 3 studies [19–21] showed that the XYC group reduced the level of serum NT-pro-BNP [RR = −126.91, 95% CI (−231.51, −22.31), *I*^2^ = 69%] (Figure 6). Due to the small number of literature available, sensitivity analysis could not be carried out.

#### 3.5.2. Quality of Life

**Figure 7:**
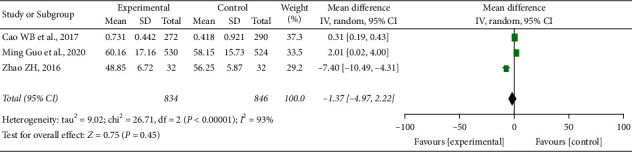
Quality of life.

Three studies [17, 22, 25] reported the quality of life of the patients, XYC group did not show better than the control group, and there was no statistically significant difference between the two groups [RR = −1.37, 95% CI (−4.97, 2.22), *I*^2^ = 93%] (Figure 7). Due to the small number of literature available, sensitivity analysis could not be carried out.

### 3.6. Adverse Reaction

Only 2 studies [22, 23] reported the adverse events of XYC, involving 636 and 629 patients, respectively in the two groups. The most common adverse drug reactions were dyspnea, palpitations, and stomach bloating. Among them, 6 patients in the XYC group had dyspnea compared to 7 patients in the control group, 2 patients in the XYC group had palpitations compared to 1 patient in the control group, and lastly, 2 patients in the XYC group had stomach bloating whereas none of the patients in the control group had stomach bloating. Compared with the control group, the total risk rate of adverse events in the XYC group was higher (1.57% versus 1.27%), which was mainly caused by stomach bloating (Table 2). The symptoms of stomach bloating can be tolerated after the addition of gastric mucosa-protective drugs.

### 3.7. Publication Biases

Because the number of studies is too insufficient to create a funnel plot, we cannot assess publication bias.

### 3.8. GRADE

The quality of evidence was assessed by two operators (Zhonghui Jiang and Hua Qu) using the Grading of Recommendations Assessment, Development and Evaluation (GRADE) approach (version 3.6), and most cardiovascular events were considered moderate scores. Some results showed low or very low evidence due to large interstudy heterogeneity and the small number of studies (Table 3).

## 4. Discussion

To our knowledge, this is the first meta-analysis to evaluate the efficacy and safety of XYC in the treatment of CAD after PCI. All 9 eligible studies were based on Chinese participants. As far as we know, XYC is mainly conducted in China, and there are no relevant trials evaluating XYC in other countries except China. In this meta-analysis, it was found that XYC had potential advantages in decreasing the occurrence of cardiovascular events (nonfatal myocardial infarction, revascularization, and readmission due to ACS) as well as improving cardiac function (LVEF, LVEDV, and LVESV) in patients after PCI. XYC can also reduce the serum NT-pro-BNP. However, XYC was ultimately found to have little effect on cardiovascular death, stroke, heart failure, and quality of life.

There are several limitations in our meta-analysis that need to be noted. First, due to the limited number of studies and patients included in these studies, the results of the meta-analysis may have certain limitations in persuasiveness. Second, it is not clear whether the efficacy of XYC is related to the interaction of conventional drugs, due to the existence of conventional drug therapy in treatments and the differences in conventional drug therapies among different studies. Third, some studies lacked specific descriptions of their research design, the generation of random sequences, randomization concealment, measurement of main results, blind methods, etc. Fourth, the adverse reactions of XYC were rarely described. Fifth, there are few studies on sample size calculation. In addition, the patients included in our systematic review were all from China, so it was difficult to determine the influence of race and region.

Acute myocardial infarction (AMI) is the most common CVD, and an estimated 7 million people worldwide suffer each year [26]. As a result of this global health burden, there has been a considerable strain on the global economy. Coronary heart disease mortality has been steadily declining in most developed countries over the past 30 years. This is primarily due to vascular reconstructive surgeries such as PCI and coronary artery bypass grafting (CABG), as well as significant advances in pharmacological treatment [27, 28]. However, the occurrence of cardiac adverse events after PCI is still a problem that cannot be ignored, which seriously affects the quality of life and prognosis of patients. Therefore, it is essential and significant to prevent cardiac adverse events after PCI. Several studies [29–31] have found that the active ingredients contained in XYC have antimyocardial ischemia, antiatherosclerosis, and antiarrhythmic effects, while regulating blood lipids, stabilizing plaque, and lowering blood pressure. Meanwhile, relevant studies have also shown that it can improve ventricular remodeling, protect cardiac function, and reduce ischemia-reperfusion injury [32–35]. According to our results, XYC may have a protective effect on the recurrence of cardiovascular events, cardiac function, and NT-pro-BNP in patients after PCI, which is consistent with the above studies.

Although this study has some limitations, its significance for clinical application and future research should be paid more attention. In future studies, we suggest that the trial protocols should be registered before conducting clinical studies, the trial design should strictly follow the principle of randomization and be double-blinded, the sample size should be strictly calculated, and the safety and adverse effects of the drug should be reasonably evaluated. Since the patients included in the systematic review were all from China, future studies should be international, and outpatients should also be considered. In the future, large samples and high-quality RCTs are still needed to further demonstrate the efficacy and safety of XYC, and we will continue to follow up this study. In the future, high-quality RCTs with large samples, rigorous design, and accurate reporting will be needed to enhance the power of evidence for the efficacy and safety of XYC. Thus, follow-up studies will be conducted.

## 5. Conclusions

XYC has potential advantages in reducing the occurrence of cardiovascular events after PCI, improving both cardiac function and NT-pro-BNP. However, some studies have shown a significant risk of bias. Therefore, in clinical treatment, it is suggested that doctors should reasonably adopt treatment strategies according to the specific conditions of patients.

## Figures and Tables

**Table 1:** Characteristics of included XYC RCTs for CAD after PCI.

Study ID	Sample size (T/C)	Age (year, T/C)	Interventions (T/C)	Duration	Outcomes
Cao et al. [17]	272/290	(58.92 ± 11.21)/(59.11 ± 11.36)	XYC + CWMT/CWMT	6 months	①②④
Chen et al. [18]	45/45	(62.03 ± 12.23)/(61.25 ± 13.46)	XYC + Fufang Chuanxiong capsule + CWMT/CWMT	6 months	②
Du et al. [19]	60/62	(63.20 ± 9.20)/(60.02 ± 10.10)	XYC + CWMT/CWMT	6 months	②③
Liu et al. [20]	50/50	(45∼75)/(47∼75)	XYC + CWMT/CWMT	6 months	①②③
Liu [21]	50/50	(61.68 ± 7.64)/(62.78 ± 8.60)	XYC + Fufang Chuanxiong capsule + CWMT/CWMT	6 months	①②③
Guo et al. [22]	530/524	(59.95 ± 9.41)/(60.40 ± 9.64)	XYC + CWMT/CWMT + Placebo	24 weeks	①④⑤
Wang et al. [23]	404/404	(31∼75)/(35∼75)	XYC + Fufang Chuanxiong capsule + CWMT/CWMT	6 months	①⑤
Zhang et al. [24]	40/39	(48.03 ± 7.59)/(46.83 ± 6.38)	XYC + Fufang Chuanxiong capsule + CWMT/CWMT	6 months	②
Zhao [25]	32/32	(55.75 ± 12.40)/(57.30 ± 11.89)	XYC + CWMT/CWMT + Placebo	24 weeks	②③④

T/C, treatment/control; CWMT, conventional western medicine treatment; XYC (SFDA registry number: Z20030073), two capsules orally, three times daily; Fufang Chuanxiong capsule (SFDA registry number: 0802205), two capsules orally, three times daily; ① primary cardiovascular events; ② heart function; ③ NT-pro-BNP; ④ quality of life; ⑤ adverse reaction.

**Table 2:** The adverse reaction of two included studies.

Adverse events	Treatment group (636)	Control group (629)
Dyspnea	6	7
Palpitation	2	1
Stomach bloating	2	0
Total risk rate (%)	1.57	1.27

**Table 3:** GRADE (quality of evidence) summary.

Outcomes	Illustrative comparative risks^*∗*^ (95% CI)		Relative effect (95% CI)	No. of participants (studies)	Quality of the evidence (GRADE)
	Assumed risk	Corresponding risk			
	Control group	Treatment group			
*Primary cardiovascular events*	Study population		RR 0.42 (0.31 to 0.56)	2,624 (5 studies)	⊕⊕⊕⊝moderate^a^
	108 per 1,000	46 per 1,000 (34 to 61)			
	Moderate				
	193 per 1,000	81 per 1,000(60 to 108)			

*Cardiac death*	Study population		OR 0.47 (0.13 to 1.68)	2,624 (5 studies)	⊕⊕⊕⊝moderate^a^
	5 per 1,000	3 per 1,000 (1 to 9)			
	Moderate				
	3 per 1,000	1 per 1,000 (0 to 5)			

*Nonfatal myocardial infarction*	Study population		OR 0.26 (0.1 to 0.7)	2,624 (5 studies)	⊕⊕⊕⊝moderate^a^
	14 per 1,000	4 per 1,000 (1 to 10)			
	Moderate				
	17 per 1,000	4 per 1,000 (2 to 12)			

*Revascularization*	Study population		OR 0.38 (0.24 to 0.61)	2,424 (3 studies)	⊕⊕⊝⊝low^a, c^
	54 per 1,000	21 per 1,000 (14 to 34)			
	Moderate				
	55 per 1,000	22 per 1,000 (14 to 34)			

*Heart failure*	Study population		OR 0.53 (0.24 to 1.2)	1,570 (4 studies)	⊕⊕⊕⊝moderate^a^
	21 per 1,000	11 per 1,000 (5 to 26)			
	Moderate				
	47 per 1,000	25 per 1,000 (12 to 56)			

*Stroke*	Study population		OR 0.52 (0.23 to 1.2)	2,624 (5 studies)	⊕⊕⊕⊝moderate^a^
	11 per 1,000	6 per 1,000 (3 to 14)			
	Moderate				
	20 per 1,000	11 per 1,000 (5 to 24)			

*Rehospitalization due to ACS*	Study population		OR 0.48 (0.33 to 0.68)	2,424 (3 studies)	⊕⊕⊝⊝low^a, c^
	80 per 1,000	40 per 1,000 (28 to 56)			
	Moderate				
	83 per 1,000	42 per 1,000 (29 to 58)			

LVEF	The mean LVEF in the intervention groups was 7.68 higher (6.85 to 8.5 higher)			1,117 (7 studies)	⊕⊕⊝⊝low^a, b^
LVEDV	The mean LVEDV in the intervention groups was 4.07 lower (5.61 to 2.54 lower)			974 (5 studies)	⊕⊕⊕⊝moderate^a^
LVESV	The mean LVESV in the intervention groups was 3.94 lower (4.94 to 2.93 lower)			974 (5 studies)	⊕⊕⊝⊝low^a, b^
IVST	The mean IVST in the intervention groups was 0.09 lower (0.4 lower to 0.22 higher)			269 (3 studies)	⊕⊕⊝⊝low^a, c^
LVPWT	The mean LVPWT in the intervention groups was 0.13 lower (0.73 lower to 0.46 higher)			190 (2 studies)	⊕⊕⊝⊝low^a, c^
LVEDD	The mean LVEDD in the intervention groups was 3.18 lower (4.16 to 2.21 lower)			169 (2 studies)	⊕⊝⊝⊝very low^a, b, c^
NT-pro-BNP	The mean NT-pro-BNP in the intervention groups was 105.61 lower (151.23 to 60 lower)			322 (3 studies)	⊕⊝⊝⊝very low^a, b, c^
Quality of life	The mean quality of life in the intervention groups was 0.31 higher (0.19 to 0.43 higher)			1680 (3 studies)	⊕⊝⊝⊝very low^a, b, c^

*∗*The basis for the assumed risk (e.g., the median control group risk across studies) is provided in the following. The corresponding risk (and its 95% confidence interval) is based on the assumed risk in the comparison group and the relative effect of the intervention (and its 95% CI). CI: confidence interval; OR: odds ratio. GRADE working group grades of evidence. High quality: further research is very unlikely to change our confidence in the estimate of the effect. Moderate quality: further research is likely to have an important impact on our confidence in the estimate of the effect and may change the estimate. Low quality: further research is very likely to have an important impact on our confidence in the estimate of the effect and is likely to change the estimate. Very low quality: we are very uncertain about the estimate. ^a^The random and blind of some studies were not clear. ^b^The interstudy heterogeneity is greater. ^c^The number of studies is small.

## Abbreviations

CAD: Coronary artery disease
PCI: Percutaneous coronary intervention
MACEs: Major adverse cardiac events
XYC: Xinyue capsule
PQS: Panax quinquefolius saponin
CENTRAL: Cochrane Central Register of Controlled Trials
CNKI: China National Knowledge Infrastructure database
RCTs: Randomized controlled trials
ACS: Acute coronary syndrome
LVEF: Left ventricular ejection fraction
LVEDV: Left ventricular end-diastolic volume
LVESV: Left ventricular end-systolic volume
LVEDD: Left ventricular end-diastolic dimension
IVST: Interventricular septum thickness
LVPWT: Left ventricular posterior wall thickness
CI: Confidence interval
RR: The relative risk
PRISMA: Preferred reporting items for systematic reviews and meta-analyses.

## Supplementary Materials

Supplementary Materials Appendix 1: search strategies for the English language databases. Appendix 2: search strategies for the Chinese databases.
