# Supplementary material for: Efficacy and Safety of Xinyue Capsule for Coronary Artery Disease after Percutaneous Coronary Intervention: A Systematic Review and Meta-Analysis of Randomized Clinical Trials
Source: Evid Based Complement Alternat Med. 2021 Apr 8;2021:6695868. doi: 10.1155/2021/6695868 (PMC8052157; doi:10.1155/2021/6695868)
Supplement: Supplementary Materials — Appendix 1: search strategies for the English language databases. Appendix 2: search strategies for the Chinese databases. [file 6695868.f1.zip › 6695868.f1/Appendix 1.doc]

## Appendix 1. Search strategies for the English language databases

**PubMed**

#1 ((((((((((Coronary Intervention, Percutaneous) OR Coronary Interventions, Percutaneous) OR Intervention, Percutaneous Coronary) OR Interventions, Percutaneous Coronary) OR Percutaneous Coronary Interventions) OR Percutaneous Coronary Revascularization) OR Coronary Revascularization, Percutaneous) OR Coronary Revascularizations, Percutaneous) OR (Percutaneous Coronary Revascularizations) OR Revascularization, Percutaneous Coronary) OR Revascularizations, Percutaneous Coronary) OR percutaneous coronary intervention OR Percutaneous Coronary Intervention OR PCI[All Fields]

#2 ((((((((((((((((((((((Artery Disease, Coronary) OR Artery Diseases, Coronary) OR Coronary Artery Diseases) OR Disease, Coronary Artery) OR Diseases, Coronary Artery) OR Coronary Arteriosclerosis) OR Arterioscleroses, Coronary) OR Coronary Arterioscleroses) OR Atherosclerosis, Coronary) OR Atheroscleroses, Coronary) OR Coronary Atheroscleroses) OR Coronary Atherosclerosis) OR Arteriosclerosis, Coronary) OR Coronary Diseases) OR Disease, Coronary) OR Diseases, Coronary) OR Coronary Heart Disease) OR Coronary Heart Diseases) OR Disease, Coronary Heart) OR Diseases, Coronary Heart) OR Heart Disease, Coronary) OR Heart Diseases, Coronary) OR CHD OR Coronary Artery Disease OR CAD[All Fields]

#3 ((((((((((((((((((Xinyue) OR Chinese Herbal) OR Ginseng) OR Ginsengs) OR Panax) OR Schinseng) OR Schinsengs) OR Renshen) OR Renshens) OR Jen Shen) OR Jen Shens) OR Shen, Jen) OR Shens, Jen) OR Shinseng) OR Shinsengs) OR Panax ginseng) OR Panax ginsengs) OR ginsengs, Panax) OR Panax quinquefolius saponin OR Xinyue capsule[All Fields]

#4 #1 AND #2 AND #3

#5 randomized controlled trial[pt]

#6 randomized clinical trial[pt]

#7 controlled clinical trial[pt]

#8 randomized[tiab]

#9 placebo[tiab]

#10 drug therapy[sh]

#11 randomly[tiab]

#12 trial[tiab]

#13 groups[tiab]

#14 #5 OR #6 OR #7 OR #8 OR #9 OR #10 OR #11 OR #12 OR #13

#15 #4 AND 14

**EMBASE**

#1 'Percutaneous Coronary Intervention'/exp OR Percutaneous Coronary Intervention

#2 'Coronary Heart Disease'/exp OR Coronary Heart Disease

#3 'Coronary Artery Disease'/exp OR Coronary Artery Disease

#4 #1 AND #2 OR #3

#5 'Xinyue capsule'/exp OR Xinyue capsule

#6 'Panax quinquefolius saponin'/exp OR Panax quinquefolius saponin

#7 #5 OR #6

#8 #4 AND #7

#9 'clinical trial'

#10 #8 AND #9

**Cochrane Library**

#1 MeSH descriptor: [Xinyue capsule] explode all trees

#2 MeSH descriptor: [Panax quinquefolius saponin] explode all trees

#3 (ginsengs, Panax):ti,ab,kw

#4	(Panax ginsengs):ti,ab,kw

#5	(Panax ginseng):ti,ab,kw

#6	(Shinsengs):ti,ab,kw

#7	(Shinseng):ti,ab,kw

#8	(Shens, Jen):ti,ab,kw

#9	(Shen, Jen):ti,ab,kw

#10	(Jen Shens):ti,ab,kw

#11	(Jen Shen):ti,ab,kw

#12	(Renshens):ti,ab,kw

#13	(Renshen):ti,ab,kw

#14	(Schinsengs):ti,ab,kw

#15	(Schinseng):ti,ab,kw

#16	(Panax):ti,ab,kw

#17	(Ginsengs):ti,ab,kw

#18	(Ginseng):ti,ab,kw

#19	(Chinese Herbal):ti,ab,kw

#20	#1 or #2 or #3 or #4 or #5 or #6 or #7 or #8 or #9 or #10 or #11 or #12 or #13 or #14 or #15 or #16 or #17 or #18 or #19

#21	MeSH descriptor: [Percutaneous Coronary Intervention] explode all trees

#22	(percutaneous coronary intervention):ti,ab,kw

#23 MeSH descriptor: [Coronary Artery Disease] explode all trees

#24	(Coronary Heart Disease):ti,ab,kw

#25 #21 or #22 or #23 or #24

#26 #20 and #25
