# Supplementary material for: Efficacy and Safety of Xinyue Capsule for Coronary Artery Disease after Percutaneous Coronary Intervention: A Systematic Review and Meta-Analysis of Randomized Clinical Trials
Source: Evid Based Complement Alternat Med. 2021 Apr 8;2021:6695868. doi: 10.1155/2021/6695868 (PMC8052157; doi:10.1155/2021/6695868)
Supplement: Supplementary Materials — Appendix 1: search strategies for the English language databases. Appendix 2: search strategies for the Chinese databases. [file 6695868.f1.zip › 6695868.f1/Appendix 2.doc]

## Appendix 2. Search strategies-Chinese databases

**CNKI**

全文=冠心病 and (全文=支架 or 全文=介入治疗 or 全文=介入手术) and 全文=心悦

**VIP**

U=冠心病*（U=支架+U=介入治疗+U=介入手术）*U=心悦

**Wanfang**

全文=冠心病 and (全文=支架 or 全文=介入治疗 or 全文=介入手术) and 全文=心悦
